# Cardiopulmonary Resuscitation Training in High School Using Avatars in Virtual Worlds: An International Feasibility Study

**DOI:** 10.2196/jmir.1715

**Published:** 2013-01-14

**Authors:** Johan Creutzfeldt, Leif Hedman, LeRoy Heinrichs, Patricia Youngblood, Li Felländer-Tsai

**Affiliations:** ^1^Department of Clinical Science, Intervention and TechnologyKarolinska InstitutetStockholmSweden; ^2^Department of PsychologyUmeå UniversityUmeåSweden; ^3^Innovation in Learning, Inc.Los Altos Hills, CAUnited States; ^4^Division of Clinical AnatomyDepartment of SurgeryStanford University School of MedicinePalo Alto, CAUnited States

**Keywords:** Serious games, virtual learning environments, MMVW, avatars, students, cardiopulmonary resuscitation, patient simulation, self-efficacy, concentration

## Abstract

**Background:**

Approximately 300,000 people suffer sudden cardiac arrest (SCA) annually in the United States. Less than 30% of out-of-hospital victims receive cardiopulmonary resuscitation (CPR) despite the American Heart Association training over 12 million laypersons annually to conduct CPR. New engaging learning methods are needed for CPR education, especially in schools. Massively multiplayer virtual worlds (MMVW) offer platforms for serious games that are promising learning methods that take advantage of the computer capabilities of today’s youth (ie, the digital native generation).

**Objective:**

Our main aim was to assess the feasibility of cardiopulmonary resuscitation training in high school students by using avatars in MMVM. We also analyzed experiences, self-efficacy, and concentration in response to training.

**Methods:**

In this prospective international collaborative study, an e-learning method was used with high school students in Sweden and the United States. A software game platform was modified for use as a serious game to train in emergency medical situations. Using MMVW technology, participants in teams of 3 were engaged in virtual-world scenarios to learn how to treat victims suffering cardiac arrest. Short debriefings were carried out after each scenario. A total of 36 high school students (Sweden, n=12; United States, n=24) participated. Their self-efficacy and concentration (task motivation) were assessed. An exit questionnaire was used to solicit experiences and attitudes toward this type of training. Among the Swedish students, a follow-up was carried out after 6 months. Depending on the distributions, *t* tests or Mann-Whitney tests were used. Correlation between variables was assessed by using Spearman rank correlation. Regression analyses were used for time-dependent variables.

**Results:**

The participants enjoyed the training and reported a self-perceived benefit as a consequence of training. The mean rating for self-efficacy increased from 5.8/7 (SD 0.72) to 6.5/7 (SD 0.57, *P*<.001). In the Swedish follow-up, it subsequently increased from 5.7/7 (SD 0.56) to 6.3/7 (SD 0.38, *P*=.006). In the Swedish group, the mean concentration value increased from 52.4/100 (SD 9.8) to 62.7/100 (SD 8.9, *P*=.05); in the US group, the concentration value increased from 70.8/100 (SD 7.9) to 82.5/100 (SD 4.7, *P*<.001). We found a significant positive correlation (*P*<.001) between self-efficacy and concentration scores. Overall, the participants were moderately or highly immersed and the software was easy to use.

**Conclusions:**

By using online MMVWs, team training in CPR is feasible and reliable for this international group of high school students (Sweden and United States). A high level of appreciation was reported among these adolescents and their self-efficacy increased significantly. The described training is a novel and interesting way to learn CPR teamwork, and in the future could be combined with psychomotor skills training.

## Introduction

Sudden cardiac arrest (SCA) is one of the most common causes of death [1]. Society depends heavily on laypersons in the initial resuscitation of cardiac arrest victims. To disseminate the necessary skills, training in cardiopulmonary resuscitation (CPR) is mandatory in many school systems. However, the effectiveness of standard CPR training among nonprofessionals [2-4] and health care professionals [5] has been questioned, mainly because of poor retention of skills and low self-efficacy among the trainees. Further, current CPR programs are focused on training of the individual. If several rescuers are present, lack of team coordination could hamper the effectiveness of CPR [6,7].

There is a demand for methods that are easy to deliver, require little material resources, and are highly engaging. Although a matter of both guideline simplicity and training methods, long-term retention is one ideal target for training. In search of new and more effective CPR training solutions, several alternative methods of delivery have been suggested during the past decade [8-12]. Screen- or simulator-based methods have been developed as alternatives to traditional manikin-based skills-oriented training. These alternatives primarily employ instructive video demonstrations or full-scale simulations.

Massively multiplayer virtual world (MMVW) technology has its roots in computer games and videogames for entertainment. Since the 1990s, videogames have been used for educational purposes in various fields, including medicine. These applications have been termed *serious games* [13]. For CPR training purposes, the option of virtual worlds for training has not been studied so far. Some encouraging results from a pilot study have demonstrated the potential of using virtual worlds for initial training of trauma team leaders [14].

In a recent study, we found that a MMVW serious game for CPR training was feasible for use among medical students. Although no evidence of stimulated recall of CPR procedures was found, the participants were enthusiastic and reported increased concentration during the training. Further, the participants’ self-efficacy increased after training [15]. *Individually perceived efficacy* refers to beliefs about one’s capabilities to learn or perform behaviors at designated levels [16]. Salas and Burke [17] have argued that for effective training, the individual characteristic of trainees, of which self-efficacy is one, should be further elucidated. Self-efficacy has been emphasized as an important feature of medical simulator training [18]. Brusso et al [19] have also pointed out the importance of self-efficacy during videogame-based training.

Information and computer technology (ICT) is becoming increasingly integrated into the everyday life of the younger generations. It has been suggested that this has implications on learning [20-23]. Although new methods for teaching based on ICT are being implemented for children and adolescents in school, knowledge is limited concerning effectiveness of such training in the medical field [24]. Taking advantage of the ICT skills of today’s adolescents regarding serious games poses potential challenges [25-27]. It has been discussed that ICT-based teaching methods often face cultural challenges when implemented in a non-native context or environment [28,29]. This fact must not be ignored when initiating multinational training programs [30].

By using a deliberate practice model [31], the aim of this prospective international study (in the United States and in Sweden) was to examine the feasibility of using a MMVW for training high school students to respond appropriately to a medical emergency requiring CPR teamwork, and to examine their experiences, self-efficacy, and concentration. Comparing the results would indicate if these results are generalizable to the 2 different student populations.

## Methods

### Recruitment and Sample

The study was performed after institutional review board approval at both study locations. After obtaining informed consent, 12 high school students from the natural science program at Huddinge High School in Huddinge, Sweden, and 24 high school students from Woodside High School in Woodside, CA, USA, were recruited and enrolled on a voluntary basis. Recruitment was carried out by their teachers and through solicitation by announcements regarding the study. Before the practice, all students had participated in compulsory conventional CPR training at their respective schools within the past 6 months. Demographic data of all study participants are displayed in Table 1. There was no clear difference between groups in mean frequency of videogame play, but a higher percentage of low-frequency and high-frequency players were found in the Swedish group.

**Table 1:** Demographics of participating high school students from Sweden and the United States (N=36).

Characteristics		Country, n (%)	
		Sweden n=12	United States n=24
**Sex**			
	Female	5 (42)	15 (62)
	Male	7 (58)	9 (38)
**Grade**			
	10	12 (100)	23 (96)
	11	0	1 (4)
**Frequency of videogame play**			
	Less than once a month	5 (42)	5 (21)
	Every second week	2 (17)	11 (46)
	Once a week	1 (8)	5 (21)
	Several times every week	2 (17)	2 (8)
	Every day	2 (17)	1 (4)

### Intervention and Curriculum Content

**Figure 1:**
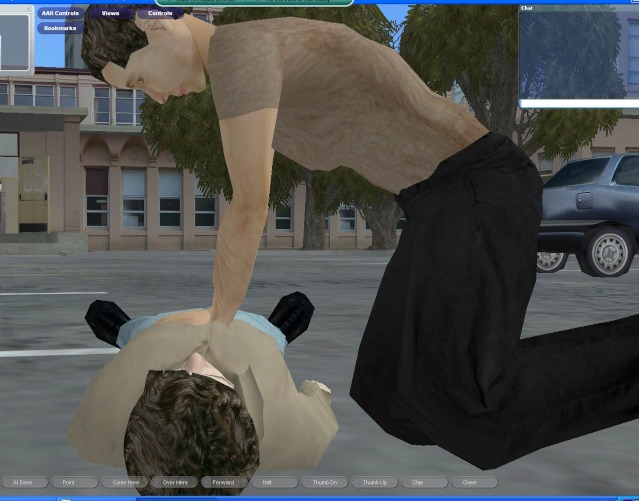
Screenshot of avatar performing chest compressions on a victim in the virtual world (parking lot scenario).

**Figure 2:**
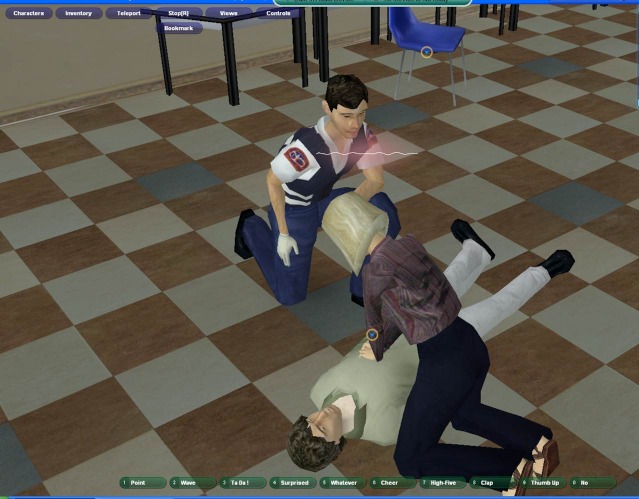
Screenshot of avatar performing chest compressions on a victim in the virtual world (classroom scenario) while talking to relieving paramedic.

The study was collaboratively planned and implemented, although it was not implemented at exactly the same time. From previous work, a virtual-world platform was jointly developed with prehospital CPR team training capabilities (Forterra Systems Inc, San Mateo, CA, USA; OLIVE game development platform now the property of SAIC, McLean, VA, USA). The virtual-world environment was tailored according to real-world examples. In the virtual world, each participant was represented by an avatar that was controlled by the computer mouse and keyboard. The participants could communicate with one another in real time by means of a microphone and headset (Figures 1 and 2).

**Figure 3:**
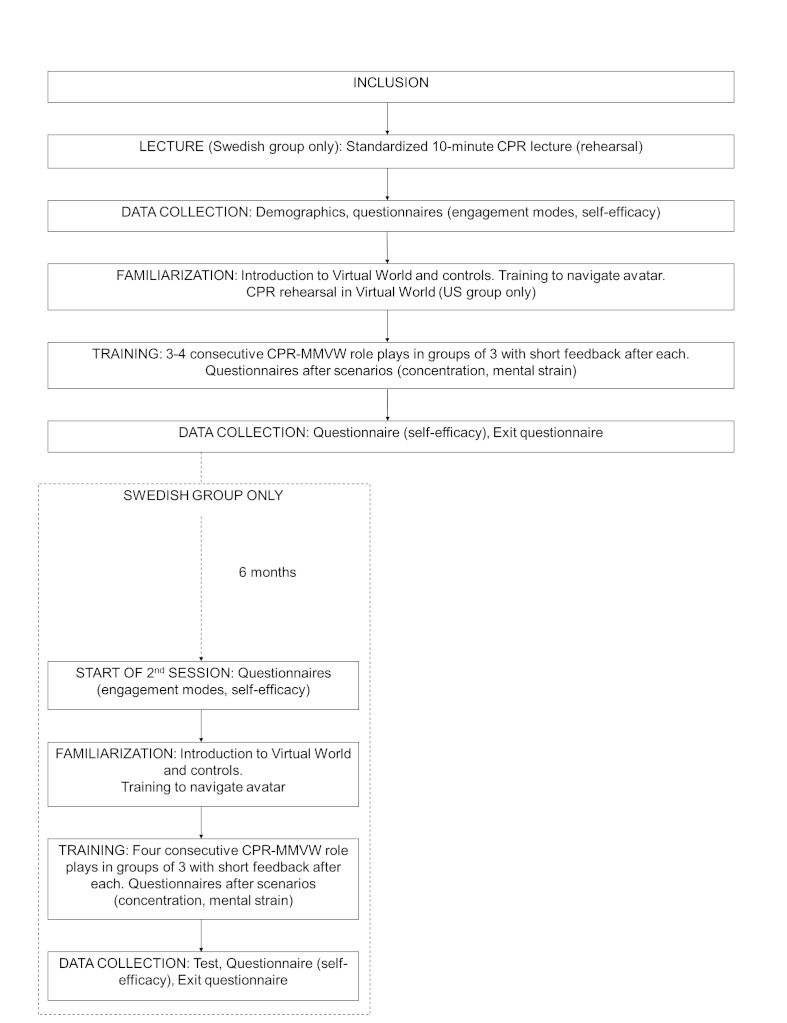
Design of the study.

An overview of the protocol is shown in Figure 3. In the beginning of the training session, the participants rehearsed and updated their CPR knowledge. In the Swedish group, this was done by a short (10-minute) lecture, whereas the US group rehearsed the CPR procedure in the virtual environment. Familiarization to the virtual world and training in maneuvering and communicating within it was done just before the CPR training started.

In the CPR scenarios, the participants trained in mixed groups of 3 students. In the Swedish study group, CPR was practiced in 4 scenarios, in which the first was regarded as a familiarization scenario. In the US group, familiarization to the virtual world included guided practice and CPR was trained in 3 scenarios. The overall time for the training sessions (including data collection) was 90 to 120 minutes.

The trainees were instructed to approach a victim (an avatar that was controlled by an instructor) whose collapse they had witnessed in the virtual world, take the correct diagnostic steps, and collaboratively perform the cognitive and procedural measures associated with basic adult life support in accordance to the 2005 guidelines from the American Heart Association [32]. These included (1) moving to the victim, (2) checking the victim for consciousness, (3) declaring the victim unconscious, (4) checking the victim’s airway and breathing, (5) calling for help, (6) performing chest compressions and rescue breaths as stated by the CPR protocol, (7) relieving the rescuers, and (8) assisting the arriving paramedic and giving a brief report to him. The settings for the virtual-world scenarios were in a classroom and in an outdoor parking lot. After each scenario, an instructor gave short feedback about their CPR performance compared to the guidelines.

Initially a follow-up was planned in both locations, but it had to be abandoned in the United States because of difficulties reassembling the study group. In the Swedish study group, a prospective test-retest explorative design could be used. This included a second session 6 months (177-203 days) after the first training. Based on the literature, 6 months was chosen as the time between the training sessions [33]. The second session was identical to the first except that the lecture on CPR was excluded. No participants in the Swedish group dropped out between these 2 training sessions.

### Instruments and Measurements

Self-efficacy, a construct resting on Bandura’s social cognitive theory [16], has been found to be a strong predictor of actual performance. Because we recently found that medical students’ self-efficacy increased after training CPR in a MMVW serious game [15], we examined if these results could be replicated in a group of young laypersons (ie, high school students). Self-efficacy was self-assessed before and after each training session by using a 5-item questionnaire in which each item was rated on a 7-grade Likert-type scale. The value for self-efficacy was calculated as the mean of these items as described elsewhere [34].

In the same study [15], medical students’ concentration increased during training. Concentration is theoretically well connected to the concept of intrinsic motivation in performing a task. A higher level of concentration is indicative of being captured by an activity and a greater probability of continuing with it. In the present study, we assessed the high school students’ concentration as an index of their motivation in the training tasks. The greater the self-efficacy, the more active the efforts would be [16]. For assessing concentration we used 8 items from already validated instruments [35,36]. In the Swedish subgroup, concentration was self-assessed after the first, second, and fourth scenario during both sessions. In the US study group, concentration was self-assessed after the first and third scenarios.

All individuals’ perceptions regarding the feasibility of training were assessed in an exit questionnaire that was distributed after the training sessions. This included the level of perceived immersion in the virtual world, questions covering technical aspects (ie, technical difficulties, ease of use), and usefulness of the training. These assessments were made by using 5-grade Likert-type scales. Control questions about self-confidence were given and the Swedish participants were also asked if this training mode could have a role in future education; 5-grade Likert-type scales were used for both questions.

In the exit questionnaire, the Swedish participants were asked to specifically comment on the perceived strengths and weaknesses of the scenarios. Meaning-bearing units [37] were identified in these comments and consequently analyzed, classified, and categorized by one of the authors (JC). If the same answer was given several times by a single participant, it was counted as only 1 occurrence to have it justly weighted during analysis. All participants were asked if and how the training had changed their feelings/perceptions about having to act in an emergency situation. In these comments, meaning-bearing entities were also identified, analyzed, classified, and categorized by the one of the authors (JC).

Although self-efficacy beliefs exercise a powerful influence on human action [16], many other psychological processes can affect the strength of this relationship. We assumed that engagement modes might potentially affect the strength of relationship between self-efficacy beliefs and behavior differently among participants. Engagement modes are different ways of interacting with new information technologies [35] and, in this study, students’ different thoughts, feelings, and purposes they had toward CPR training using avatars in MMVM. We calculated an index of the negative engagement modes (sum of scores for the frustration/anxiety and hesitation/avoidance modes), as described by Hedman and Sharafi [36], because they may be associated with lower self-efficacy. Negative engagement modes were assessed before each session from a 15-item questionnaire using a 5-point Likert-type scale.

We assumed that very high mental strain could be another factor that may jeopardize perceived self-efficacy. Only in the Swedish subgroup, mental strain could be self-assessed after the first, second, and fourth scenario during each session. Mental strain was measured using Borgs’ CR10 scale (0 =no mental strain at all; 10 = extremely high mental strain) after each session [38].

### Data Analysis

Participants with single missing data were excluded from that particular analysis. Repeated measurements analysis was used to analyze time-dependent data (concentration) and regression analysis was used to evaluate the dependency between these variables. For correlation between concentration and self-efficacy, Spearman rank correlation (ρ) was performed. Statistical comparisons were made by using the Mann-Whitney test or the Student *t* test, after validation for normal distribution by using the Shapiro-Wilk test. The significance level was set at *P*<.05. The calculations were performed by using SigmaStat version 3.5 (Systat Software Inc, Point Richmond, CA, USA). Data are presented as mean and standard deviation (SD) or median and interquartile range (IQR) depending on the type of distribution.

## Results

Summative data from Sweden and the United States were analyzed as one group, except for concentration in which there were large differences between the groups.

### Controlled Factors

Negative engagement modes in the Swedish study group occurred during session 1 (mean 5.4, SD 1.1) and during session 2 (mean 5.4, SD 1.1). In the US study group, the mean was 3.4 (SD 0.3). The difference between the groups was significant (*P*<.001). The mean value of mental strain, measured only in the Swedish group, was low (mean 3.4, SD 1.7) without significant change over time.

### Self-efficacy and Concentration

**Figure 4:**
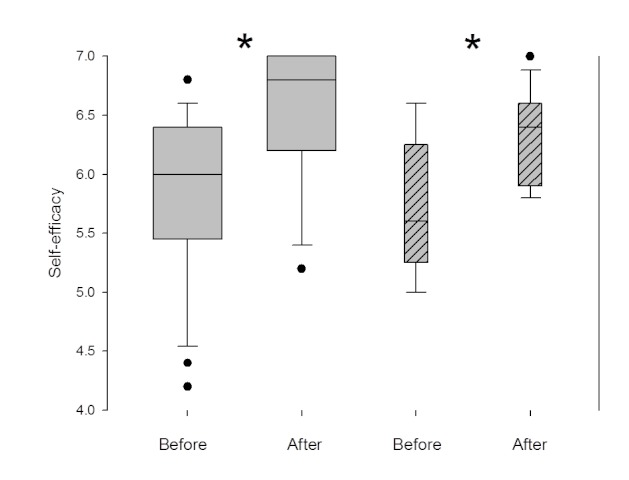
Self-efficacy in the study group. The two left-most boxes present self-efficacy before and after training (N = 36).The two right-most boxes (striped) refer to the second session during the Swedish part of the study (n = 12). The box-blot illustrates 25th and 75th percentiles with median value as a solid line inside the plot and whiskers showing 10th and 90th percentiles (outliers marked outside this). Significance (P <.05) between the measurements is denoted with an asterisk (*).

Self-efficacy increased significantly after training compared to before training (Figure 4). In the Swedish group, this was also replicated during the second training session.

In general, the concentration (task motivation) level was in the medium to high range. However, the results in the US group were significantly higher than the Swedish group (*P*<.001); in the latter, the mean concentration score showed a significant increase over time (scenario 1 of session 1: mean 52.4, SD 9.8; scenario 4 of session 2: mean 62.7, SD 8.9; *P*<.05). In the US group, the mean concentration score increased significantly from 70.8 (SD 7.9) to 82.5 (SD 4.7, *P*<.001).

Self-efficacy was positively related to concentration during training before and after training (self-efficacy before training and concentration during first scenario: ρ = 0.49, *P*=.006; self-efficacy after training and concentration during last scenario: ρ = 0.60, *P*<.001).

### Perceptions of the Training and Attitudes

The exit questionnaire contained information about the participants’ experiences of the technical aspects of the virtual world and their opinions about the MMVW CPR team training. The participants’ ratings indicated a significant change of confidence after the training (*P*<.001). Numerical ratings are summarized in Table 2. Table 3 summarizes perceived strengths and weaknesses with the simulated scenarios, investigated in the Swedish subgroup.

Twenty-eight of the 36 participants (78%) agreed that the MMVW experience had changed their feelings or perceptions about responding to a medical emergency, without any differences between study groups (Table 4).

**Table 2:** Exit questionnaire about the participants’ perceptions of the training and attitudes toward it based on 5-point Likert-type scales.

Exit question	Swedish group, median (IQR)^a^		US group, median (IQR)^a^
	Session 1 n=12	Session 2 n=12	n=24
Did you feel that you were actually there?^b^	4.0 (3.5-4.5)	4.0 (3.0-4.0)	4.0 (4.0-4.0)
Did you experience any technical difficulties?^b^	2.0 (1.5-2.5)	2.0 (1.0-2.5)	2.0 (2.0-3.0)
How easy to learn to control your avatar?^c^	4.0 (3.5-4.5)	4.0 (3.5-5.0)	4.0 (4.0-5.0)
How useful for learning to react to a medical emergency?^d^	—	4.5 (4.0-5.0)	4.0 (4.0-5.0)
Do you think this type of simulated training has a part in the education of tomorrow?^e^	5.0 (4.0-5.0)	5.0 (5.0-5.0)	—
How confident to react to a medical emergency before today’s session?^f^	—	4.0 (3.0-4.0)	2.5 (2.0-3.0)
How confident to react to a medical emergency after today’s session?^f^	—	4.2 (4.0-5.0)	4.0 (4.0-4.5)

^a^ IQR: interquartile range; —: question was not asked.

^b^ 1 = not at all; 5 = all of the time.

^c^ 1 = never learned how; 5 = very easy.

^d^ 1 = not useful; 5 = very useful.

^e^ 1 = not at all; 5 = yes, absolutely.

^f^ 1 = not confident; 5 = extremely confident.

**Table 3:** The Swedish participants’ (n=12) answers about strengths and weaknesses of the simulated scenarios.

Category		n (%)
**Strengths**		
	Suitable and realistic environment	9 (28)
	Good way to repeatedly practice and learn	8 (25)
	Necessary to adapt to changing circumstances	2 (6)
	Training teamwork aspects	7 (22)
	Good in general	6 (19)
**Weaknesses**		
	Too easy tasks, more options wanted	10 (30)
	Lack of realism and a richer environment	8 (24)
	Technical problems	9 (27)
	Requires familiarization	6 (18)

**Table 4:** Statements about how the training changed the participants’ (n=36) feelings or perceptions about responding to a medical emergency (participants could give several answers).

Category	n (%)
Work better in a CPR team	11 (26)
Increased confidence for such an emergency situation	20 (47)
Better CPR knowledge	12 (28)

## Discussion

Our ambition in this feasibility study was to selectively analyze how teams of young laypersons, personified as avatars, reacted toward and interacted within a virtual world for teaching how to respond appropriately to a medical emergency requiring CPR. Our study sample were high school students in an international setting. In this study, we refined and used a virtual world for CPR team training previously studied in medical students [15]. The high school students clearly appreciated the way the training could influence real-world behaviors in several aspects and it was associated with an increased self-efficacy in both countries. Our results are consistent with previous findings in a group of medical students [15]. These findings suggest that this completely novel method for CPR training is also feasible and reliable in young laypersons.

The increase in students’ self-efficacy has 2 important implications. First, it illustrates the beliefs of the trainee (ie, that the level of control of required skills increased). We triangulated this finding with the control questions in the exit questionnaire about the participants’ self-confidence that also increased because of the training. Secondly, which may be particularly important concerning CPR teamwork, it gives an indication that the trainee actually would feel more prepared to act in a real-world CPR event. This assumption should be tested in future studies.

Our finding that there was a positive correlation between self-efficacy and concentration (as an index of motivation in the training tasks) is in accordance with Bandura’s [16] social cognitive theory in which self-efficacy beliefs influence cognitive, motivational, affective, and decisional processes. The course-effect relationship between self-efficacy and concentration should, however, be elucidated by using a control group design. Concentration and the perception of immersion, characteristics of computer games, are of great importance because these are signs of how the trainees are engaged in the training. A high level of task involvement is a prerequisite for active learning. Concentration has been positively associated with effective teaching and learning [39]. Current CPR training seems to carry problems in these aspects, which might lead to a lower degree of influence on the trainees and, in turn, less retention.

An important aspect is the degree of realism and sense of presence in virtual-world training. There certainly is a risk that the participants may perceive the training environment as awkward and experience a lack of real-world resemblance. In the past few years, virtual-world training has been used extensively for cybertherapy in psychiatry in which these aspects are crucial. Studies from this field show that this type of training has potential for immersion and active engagement [40,41]. Also, our results indicate that the learning scenarios and technology developed for our study were effective for most students in achieving a “suspension of disbelief.”

Although the need for CPR training is universal, computer habits as well as pedagogical approaches and attitudes toward e-learning differ greatly among different countries and cultures [42,43]. Attitudes toward team interactions in virtual settings seem to be of great importance and may also differ. Ahanchian and McCormick [28] created a framework based on Hofstede’s cultural dimensional framework to assist understanding of cultural differences. With that in mind, we expanded the study to another contextual and cultural setting to analyze the generalizability of MMVW as a new teaching method for team-based CPR training. Videogame habits among the participants in the 2 different countries were somewhat different. In particular, the Swedish group contained a larger group of participants with less computer gaming activity. Although there is some variation in the literature, videogame habits among the participants did not seem to be above average compared to larger US samples [44,45]. Wong [29] has pointed out potential difficulties with e-learning in multicultural settings, in which cultural influences may impact how the learning is perceived and its effectiveness. These problems primarily have their roots in cultural-sensitive issues, such as attitudes toward how one should behave in an e-learning situation or how active and self-directed the learner is expected to be. In the present MMVW, the only interaction between participants was by direct voice interaction in the virtual world, and the interaction with the teacher or instructor was either in-world (by the use of avatars) or in the real classroom. Hence, the virtual world itself will not be affected by cultural issues, except for appearances of objects (eg, buildings, avatars) and the text on the interactive buttons used to examine and treat the victim. On the other hand, Uzuner [46] pointed out in a review that attitudes toward distance learning may differ greatly among different cultures, in particular between so-called collectivistic and individualistic cultures. Without further data from our international study, we cannot draw any conclusion about how culture influences our findings. However, the aim was to study if our findings would be similar in the 2 countries and, hence, more generalizable. As with leisure gaming MMVW, our data quite clearly indicate that users in different countries enjoy similar engagement in this MMVW CPR team training.

The major difference in the individual experiences during training between the 2 countries was in concentration, where the US sample was on a considerably higher level, in general. In this group, the negative engagement modes in how one relates to information technology was also considerably lower. This difference in attitude toward the training method, indicating a more relaxed and open attitude toward ICT in the US sample and reflecting the smaller percentage of participants with low-frequency computer gaming activity, may explain some of the difference in concentration. Another reasonable explanation could be that the educational software was more developed and stable, and technical and educational support was more easily accessible at that time. During the training in the United States, developers and technical expertise was readily present.

The individual comments indicate that MMVW team CPR training was appreciated for its team aspects and for being easy to practice repetitively leading to better knowledge and confidence. On the other hand, among the Swedish participants, remarks were made about the tasks being too easy and with too few options for interaction. Such indications warrant further investigation. One of the appealing features of serious games are their ability to vary the level of difficulty and keep the user in a high level of engagement—these comments indicate that more could be accomplished in these aspects, possibly leading to higher levels of concentration.

The results from these 2 groups are in accordance with what we have found when studying a group of medical students [15]. This implies the feasibility of the concept that MMVW for training CPR teamwork can be carried out easily and with good acceptance from the trainees. Personal observations, reviews of ratings, and verbal and written comments indicate that this new learning technology is highly appreciated by high school participants. Although CPR was the training content for this study, virtual worlds may be used for other medical training purposes. Patel and coworkers [47] have used an interactive virtual-world session for introducing the operating room environment to novices. Compared to standard real-world introduction, they found the virtual-world introduction to be as beneficial for gaining knowledge, skills, and attitudes. However when it comes to training of team activities, there are few reports in the medical literature.

Important issues when designing CPR training methods are cost and adaptability [48,49]. Not only is it important to find an engaging and popular form of training, it must also be associated with reasonable costs and carry the advantages of being easily adopted to different target groups as well as future changes in CPR guidelines. Further, it is also of great importance that the system and method used is easy to operate from a teacher’s perspective. A complex method requiring high computer interest and skills from the teacher’s side can be expected to be difficult to implement.

In society, initiation of action in sudden emergency medical situations depends on the vigilance of bystanders. The problem of delivering up-to-date education and training in this field for all citizens is huge. To deal with retention problems through recurrent training is perhaps even more challenging. Although some evidence of good retention after conventional training in school-aged children exists [50], many reports are discouraging.

Today, manikin-based CPR training is the norm. Such training carries several weaknesses (eg, accessibility, cost, engagement among trainees, lack of team aspects, and low long-term efficacy). Some of these issues may be addressed by the use of MMVW CPR team training. Further, the uniqueness of this training not only lies in the different medium for interaction and transmission, but also in the possibility to train CPR in a “live” setting where the medical emergency is put into context. Also, flexibility in changing the virtual environment, number of rescuers, and level of difficulty and complexity is increased compared to standard CPR training. On the other hand, using virtual worlds for training creates new demands, such as computer hardware and Internet connections, trainee computer skills, and time for learning the software and familiarizing to the virtual environment.

Current pedagogical training concepts advocate the division of complex tasks into the training of its included parts during basic training [51]. CPR consists of cognitive as well as team and psychomotor skills. Hence, it might be beneficial to learn and train the different aspects of CPR in different modes and at different times. When the separate parts are mastered, they may be integrated and trained. In its current format, MMVW CPR only consists of some parts of the whole, cognitive, and team aspects, whereas the psychomotor skills are not covered. To cover the full extent of bystander CPR (or other forms of CPR), this training modality has to be developed further.

The main goal for this study was not to validate or test a particular virtual world, but rather to explore the concept and study feasibility aspects. Although manikin-based training continues to be the prevailing method for learning the basic psychomotor skills of CPR, this study demonstrates the potential added value of MMVW for situated and team-based learning in which laypersons are able to practice the sequence of actions necessary to respond appropriately to different medical emergencies. One of the most obvious advantages with virtual-world training is its almost endless variability. Changes in virtual environment, equipment, and situation creates potential means for practicing a wide variety of medical tasks, many that normally would be difficult to practice in the real world.

A limitation of this work is the sample size. The originally planned follow-up in the United States was abandoned because of a dropout rate of almost 50%. This occurred because the follow-up had to be scheduled in the subsequent school year. Students had either left the district, changed schools, or were in classes in which their absence was not allowed which precluded participation in the study follow-up. It certainly turns the focus to the difficulties in carrying out multinational field studies in which adherence to initial protocol was restrained because of cultural and curriculum circumstances. In future studies, this issue has to be carefully addressed in close cooperation with the school administrators and staff. Our intention was to study the samples at a time when CPR training was part of their normal curriculum. Further, the study was carried out in schools situated in 2 high-income Western countries. Although we found the same patterns in the results in the 2 countries, these results would be easier to generalize if more groups were studied (ie, different cultures and ages). Future studies should investigate retention after training and transfer to real-world CPR. Also, more data on the teachers’ perceptions are warranted.

### Conclusions

The data from this feasibility study support the use of MMVWs for teaching high school students to respond appropriately to a medical emergency requiring CPR teamwork. It was feasible, reliable, and enjoyed by digital natives in Sweden and in the United States. This study demonstrates the potential added value of MMVW for situated learning in which young laypersons are able to practice the sequence of actions necessary to respond appropriately to different medical emergencies.

Even with a great demand for new training methods in high school and in the area of CPR training, MMVW serious games must be carefully studied not only from the students’ perspective, but also from the teachers’ and organizations’ perspectives to clarify the challenges and needs required for implementation.

## Abbreviations

CPR: cardiopulmonary resuscitation
ICT: information and computer technology
IQR: interquartile range
MMVW: massively multiplayer virtual world
SCA: sudden cardiac arrest
